# Supplementary material for: DMRforPairs: identifying Differentially Methylated Regions between unique samples using array based methylation profiles
Source: BMC Bioinformatics. 2014 May 15;15:141. doi: 10.1186/1471-2105-15-141 (PMC4046028; doi:10.1186/1471-2105-15-141)
Supplement: Additional file 2 — DMRforPairs output for the comparison of A431-MCF7 and NA17018-NA17105. Please start from the HTML files in each folder. Available via the BMC Bioinformatics website. [file 1471-2105-15-141-S2.zip › 1394847754114233_MOESM2_ESM/A431_MCF7/figures/25568.pdf]

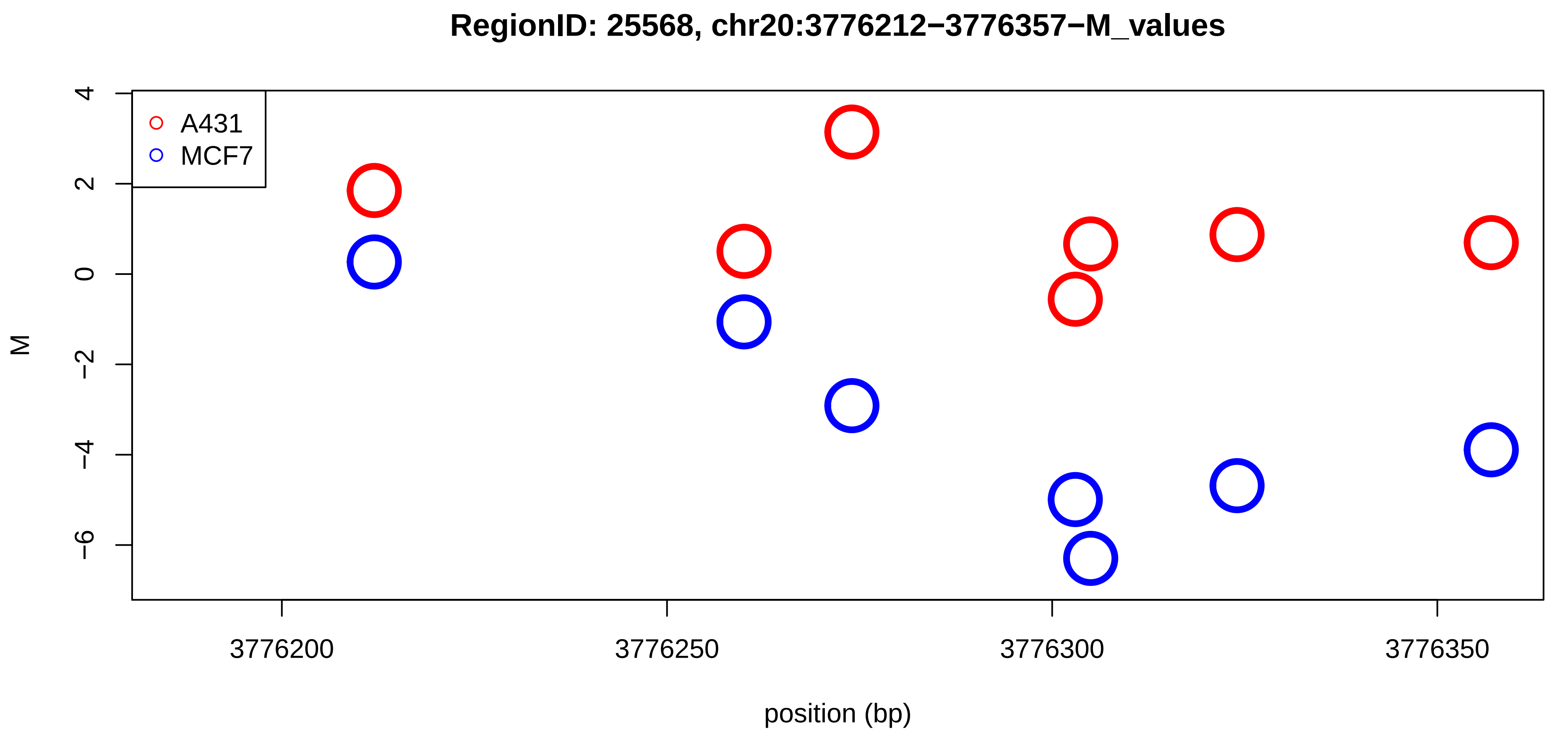

RegionID: 25568, chr20:3776212−3776357−M_values
A431
MCF7
M
4
2
0
−2
−4
−6
3776200
3776250
3776300
3776350
position (bp)

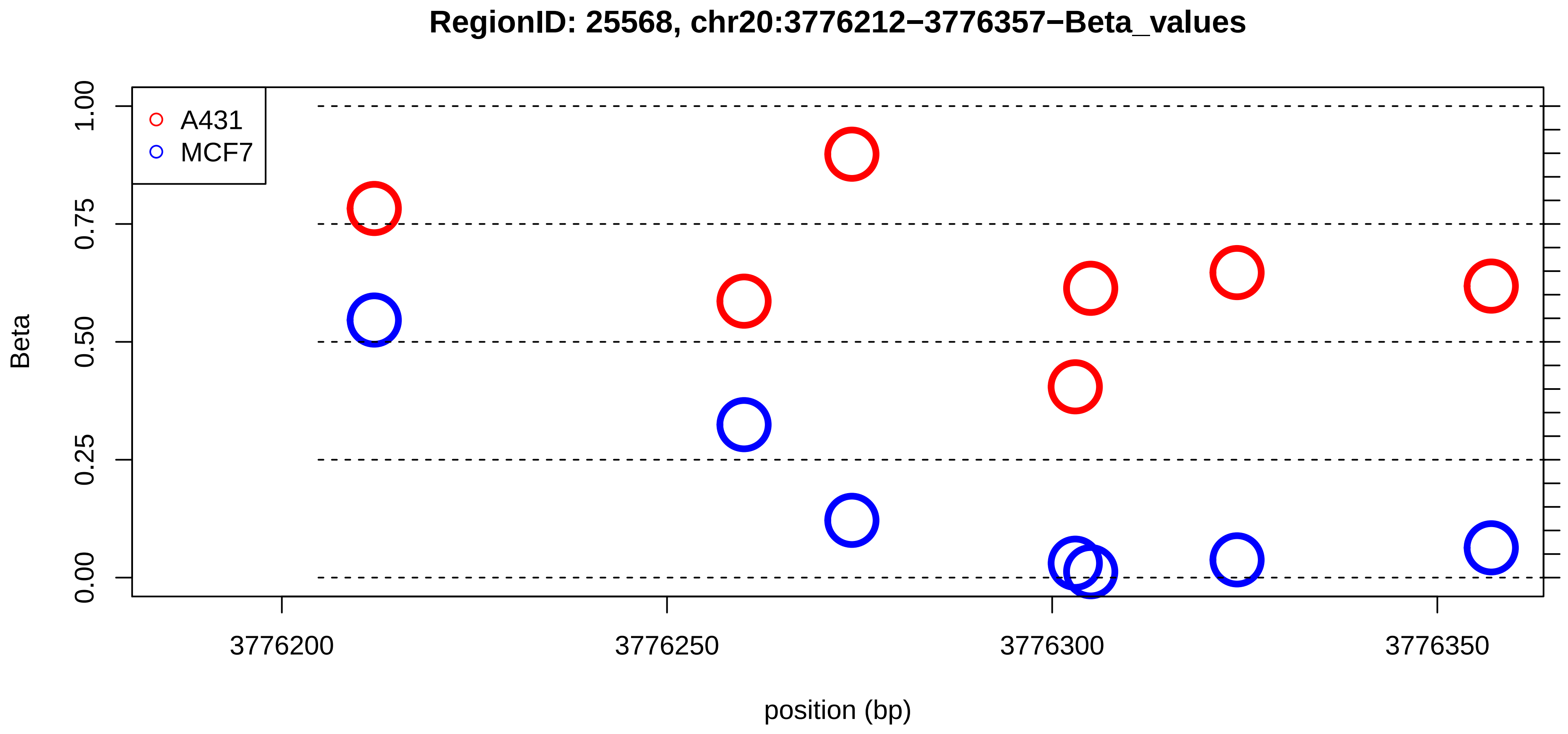

RegionID: 25568, chr20:3776212−3776357−Beta_values
A431
MCF7
Beta
1.00
0.75
0.50
0.25
0.00
3776200
3776250
3776300
3776350
position (bp)

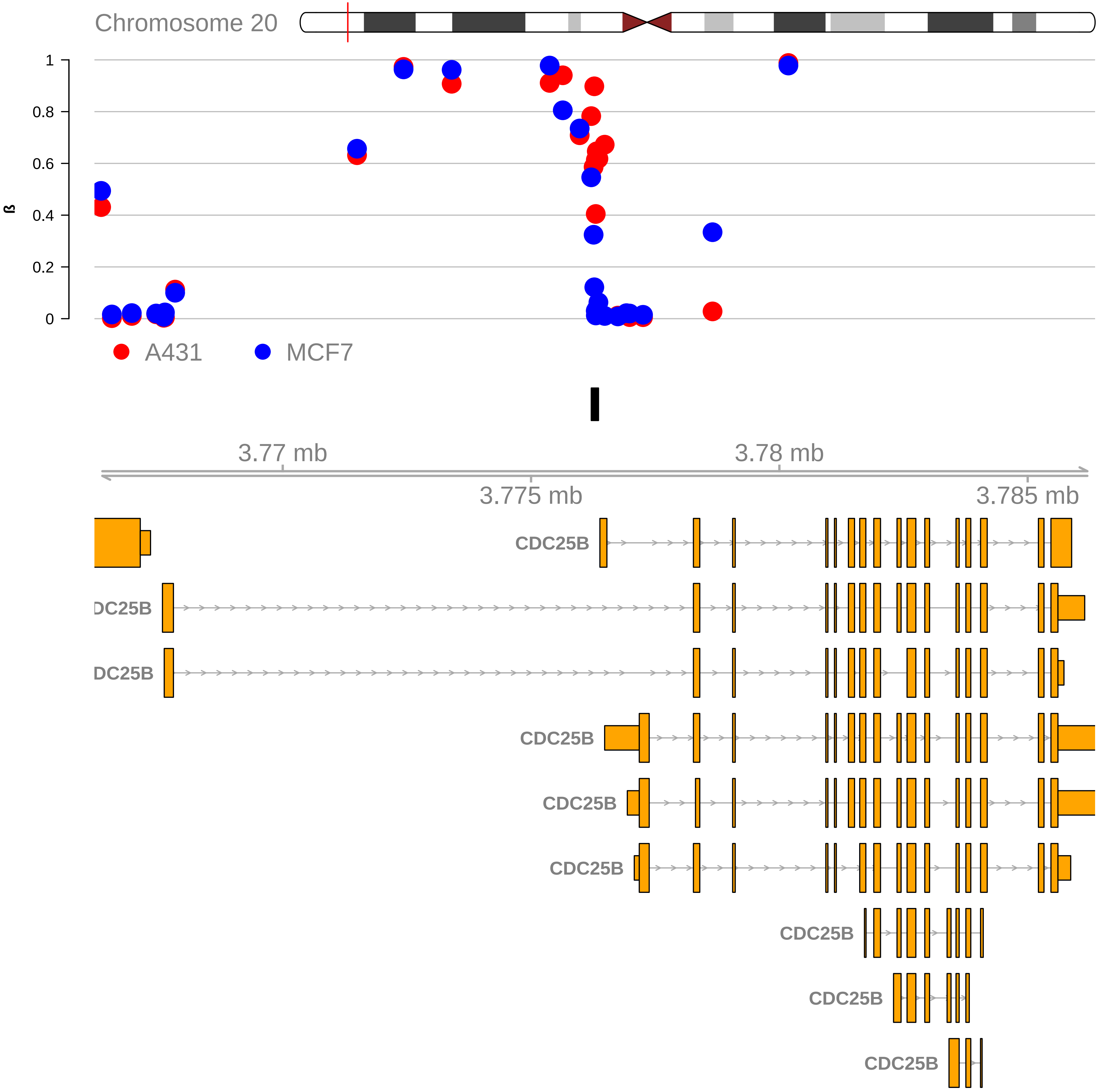

Chromosome 20
β
1
0.8
0.6
0.4
0.2
0
A431
MCF7
3.77 mb
3.78 mb
3.775 mb
3.785 mb
CDC25B
DC25B
DC25B
CDC25B
CDC25B
CDC25B
CDC25B
CDC25B
CDC25B
